# Disrupting drive-by download networks on Twitter

**DOI:** 10.1007/s13278-022-00944-2

**Published:** 2022-08-20

**Authors:** Amir Javed, Ruth Ikwu, Pete Burnap, Luca Giommoni, Matthew L. Williams

**Affiliations:** 1 School of Computer Science and Informatics, Cardiff University, Cardiff, UK; 2 School of Social Sciences, Cardiff University, Cardiff, UK

**Keywords:** Cybersecurity, Drive-by download, Malware, Machine learning, Cybercrime

## Abstract

This paper tests disruption strategies in Twitter networks containing malicious URLs used in drive-by download attacks. Cybercriminals use popular events that attract a large number of Twitter users to infect and propagate malware by using trending hashtags and creating misleading tweets to lure users to malicious webpages. Due to Twitter’s 280 character restriction and automatic shortening of URLs, it is particularly susceptible to the propagation of malware involved in drive-by download attacks. Considering the number of online users and the network formed by retweeting a tweet, a cybercriminal can infect millions of users in a short period. Policymakers and researchers have struggled to develop an efficient network disruption strategy to stop malware propagation effectively. We define an efficient strategy as one that considers network topology and dependency on network resilience, where resilience is the ability of the network to continue to disseminate information even when users are removed from it. One of the challenges faced while curbing malware propagation on online social platforms is understanding the cybercriminal network spreading the malware. Combining computational modelling and social network analysis, we identify the most effective strategy for disrupting networks of malicious URLs. Our results emphasise the importance of specific network disruption parameters such as network and emotion features, which have proved to be more effective in disrupting malicious networks compared to random strategies. In conclusion, disruption strategies force cybercriminal networks to become more vulnerable by strategically removing malicious users, which causes successful network disruption to become a long-term effort.

## Introduction

In recent years, social media platforms such as Twitter, Facebook and Instagram have increased their presence in our daily lives. As of the 2 January 2020, Twitter alone reported 145 million daily users and 500 million tweets daily (Smith 2020)—an average of 6000 tweets per second (MohamedSikandar 2018) and a highly connected complex network of users, capable of propagating news from one corner of the world to another in a matter of seconds. Unfortunately, this information dissemination power of online social networks has been exploited by infamous cybercriminal groups such as Lurk (Yarochkin 2017) and Patchwork (Lunghi and Horejsi 2017) (or Dropping Elephant) by their designing and executing of cyberattacks specific to online social platforms. Though social media has a great impact on the connectivity in our lives, it leaves us open to many forms of cyberattack (Ghosh 2019). Cyberattacks such as Phishing (Sabbagh 2020), ransomware (Sabbagh 2020) and drive-by downloads (Javed et al. 2018) are on the rise (Interpol 2020) and are being continuously adapted to be delivered through online social networks. Though the code/tactics behind drive-by download attacks are constantly changing the delivery mechanism, posting(tweeting)/sharing (retweeting) a malicious URL withing a post remains the same. Thus, making them one of the most dangerous attack on OSN that account for 48% of attacks by exploiting Web-based vulnerabilities (SANS Institue 2017).

Current research related to drive-by downloads on Twitter has focused either on its detection (Burnap et al. 2015; Huang et al. 2021; Kim et al. 2017; Zhang et al. 2021; MarkMonitor 2019; Group 2015), prediction (Javed et al. 2018) or its propagation (Sanzgiri et al. 2012). Detection models have been developed based on network traffic, machine behaviour, social characteristics of users posting URLs, static and dynamic analysis of code within a Web page. A prediction model was developed based on machine (operating system and network) and social characteristics (Javed et al. 2018). Efforts have been made to identify malicious user and understand malware propagation within these complex online social networks to curb the malware infection rate. For example, Jang et al. (2015) proposed a novel method to detect malicious applications by creating a network graph from the system calls a program makes, followed by applying social network analysis to identify malicious applications.

Malware propagation has been studied using epidemiology concepts (Sanzgiri et al. 2012), by applying game theory to understand the relationship between users propagating malware (Sun et al. 2012), by observing posting behaviour of a user on OSN (Yan et al. 2011) and by understanding factors related to virality and survival of malicious tweets (Javed et al. 2020). Considering the rate at which news disseminates within networks, detection and propagation models that have been proposed so far may not be enough to curb malware propagation. There is a need to strategically remove users from the network by implementing a network disruption strategy to decrease its connectedness to stop malware propagation. This paper proposes different strategies to disrupt malicious user networks and identify the most effective approaches to curb propagation by removing the minimum number of users. An effective strategy substantially reduces network connectedness by removing the smallest number of users so that malware exposure is limited to a few people. Ours is a novel contribution with which it is possible to strategically remove influential malicious users from the network before an attack has exposed millions, proactively preventing the propagation of an attack. This is the first study to the identify critical network nodes for the purposes of malicious network disruption and to empirically test the impact of different strategies.

## Related work

As technology has evolved, so has malware. The increasing complexity of users’ communication on the Internet has given cybercriminals several methods to propagate malware. The current research on malware propagation is broadly divided into two categories: understanding malware propagation on online social networks and disrupting malware propagation.

*Malware Propagation on Online Social Networks :-*

With the growing popularity and complexity of online social networks, cybercriminals have shifted their focus from traditional (network formed by connecting users via mobile devices (Fleizach et al. 2007), Bluetooth (Cheng et al. 2011), email (Wen et al. 2014)) to online social networks (such as Facebook (Fan and Yeung 2011) and Twitter (Sanzgiri et al. 2012)) to attack millions of users within minutes. OSN have introduced new communication methods such that new social relationships (Fleizach et al. 2007) and connections between users give cybercriminals another medium through which they can propagate malware. Research on malware propagation via these mediums has analysed users’ behaviours (Yan et al. 2011; Wang et al. 2017) and relationships (Chen et al. 2017) to shed light on the infection rate and reach of malware (Sanzgiri et al. 2012; Wang et al. 2016). Given the constant evolution of malware, propagation models have to incorporate new features to tackle new techniques used by cybercriminals. Researchers applying epidemiology concepts have demonstrated malware dissemination can be attributed to a small number of users (Sanzgiri et al. 2012) and that social network characteristics, such as degree centrality, are key to identifying propagators (Jang et al. 2015). Research has also shifted the focus from network-based features to user and content features of malicious code propagators. For example, the relationship between content-based features, such as sentiment and emotion, has been established in research detecting spam (Wang et al. 2015; Hu et al. 2014). More recently, Javed et al. have uncovered predictive features for the sharing of content on Twitter (Javed et al. 2020), discovering the emotion of fear to be statistically significant in the propagation of information flows and concluding that malicious tweet containing fear-related words were more likely to be retweeted than those did not.

*Disrupting malware propagation* :- Current research on curbing malware propagation focuses on understanding the malicious network and its actors. Research has been undertaken to understand factors influencing propagation (Javed et al. 2020) and understanding how diffusion of news relates to malware and Ransomware propagation on Twitter (Puliga12 et al. 2018). It is also imperative to understand the types of malicious actors that are currently active on Twitter by highlighting their characteristic behaviours (Jamison et al. 2019). However, the focus to date has been on understanding the propagation of malware on online social networks, with limited research on proposing disruption strategies. While extensive studies have covered the nature of online criminal networks (Willis et al. 2015; Duijn 2016) and have proposed different disruption strategies to neutralise these criminal networks, none have been undertaken on Online Social Networks (OSNs) for malware.

Having said that, online criminal networks are similar to OSNs because they are made up of multiple actors (which take the form of users in OSNs), with relationships and social interactions between these them (such as following, liking and reposting on OSNs). Previous attempts at studying cybercriminal networks have focused mainly on understanding the structure of networks and their key actors. Although most of these studies refer to subject areas outside OSNs, the results have implications for the disruption of adversarial networks in OSNs. Therefore, we have grounded our work on the literature about criminal networks, transmissible infections and malware distribution. For example, Das and Sinha used network characteristics such as centrality to remove malicious nodes that slowed the dissemination of information (Das and Sinha 2016), where they defined *malicious nodes* as those nodes that slow dissemination of information. Furthermore, Baker and Faulkner (1993) reconstructed the malicious social communication networks involved in price-fixing in the electrical equipment industry. Their research concludes that it is essential to assume that criminal operations are set up to maximise concealment rather than efficiency in considering disruption strategies. In recent times, Pedahuzu Perlinger (Perliger and Pedahzur 2011) studied the organisation of terrorist networks in OSNs, highlighting the necessity to clearly define key actors and group roles when designing mitigation strategies. Bruns (2011); Sevastopulo and Dyer (2015) explained how US intelligence service leveraged communications and account data on Twitter to track members and activities that led to the capture of Osama Bin Laden during the Obama administration. In particular, they used Twitter’s geographical location tags to track conversations related to known Bin Laden affiliates.

Gerdes (2015) identifies two areas of focus for investigating strategies for network disruption: (1) disruption as a strategy to identify and break up connections between concealed actors (removing key actors from the network), and (2) disruption as a strategy that attacks the efficiency of the network, i.e. minimising the spread or reach of key actors. Therefore, social network disruption can refer to removing nodes that result in separate subgroups and isolating nodes or removing nodes that minimise the rate of interaction with malicious material. Both forms of network dismantlement involve identifying and removing key actors within the network. However, Gerdes (2015) highlights the definition of a ‘key actor’ could present a challenge when defining its role in the network. Key actors may be defined as members who identify as part of the network or those who do not identify with these networks but are essential to achieving the maximum impact of criminal activities online. In social network analysis (SNA), most key actors essential to malicious code distribution act as hubs with a more significant number of connections relative to other nodes or as ‘brokers’ who connect the most significant number of nodes (Reid et al. 2014). Some researchers show that broker removal is effective for resilient networks that seek to maximise the attack’s impact. For instance, Keegan et al. (2010) tests the resilience of an illicit drug trafficking network masquerading as an online gaming network. The aim was to test the effectiveness of disruption strategies by measuring the network’s time to reform and continue illicit activities. They concluded that removing top-ranking nodes based on degree centrality effectively dismantles the networks. However, removing top-ranking nodes based on random removal in sequential order failed to yield similar results.

On the other hand, hub removal is effective for networks deeply connected underground with exclusive membership rights (Bright et al. 2015). Xu and Chen (2008) investigate the nature of drug trafficking and gang networks and recommend two disruption strategies for such scale-free networks. The first is the sequential removal of hubs based on maximum degree centrality, and the second removes nodes with high betweenness centrality. Their evaluation of these disruption strategies found subtle indicators favouring influence over connectivity. Finally, Bright and Delaney (2013) show that removing hubs in a deep sex trafficking network effectively reduced the size of the most significant connected component after disruption and the number of isolates after a disruption. Similarly, Musciotto and Miccichè analysed Cosa Nostra criminal network and applied a network disruption strategy based on the degree centrality and betweenness to dismantle a criminal network (Musciotto and Miccichè 2022). They used a heuristic approach for their analysis which was not based entirely on network features that usually are not available without having a full description of the system, but on properties that can be measured effectively in earlier phases of analysis. This demonstrated that an effective interruption is possible at an early stage to dismantle a criminal organisational network. Furthermore, network parameters such as degree and betweenness centrality have been used in identifying individuals who are likely to play critical roles in the dissemination of transmitted diseases (Hsieh et al. 2014). Thus, these parameters have played a key role in creating network disruption strategies to dismantle/disrupt criminal networks (Giommoni et al. 2021) or curb transmission of infectious diseases.

With the evidence of infamous cybercriminal groups such as Lurk (Yarochkin 2017) and Patchwork (Lunghi and Horejsi 2017) (or Dropping Elephant) executing cyberattacks specific to online social platforms and these web-based attacks account for 48% of attacks by exploiting Web-based vulnerabilities (SANS Institue 2017). Therefore, there is a need to understand this criminal network and design effective network disruption strategies to disrupt malware propagation. Research has been done in understanding malware propagation by developing models based on epidemiology and graph theory (Sanzgiri et al. 2012; Liu et al. 2016; Ganesh et al. 2005; Jyothi and Vorugunti 2017). To the best of our knowledge, there has not been any study on network disruption of malicious users network to curb malware propagation. However, research has been done in understanding and disrupting criminal network by designing simple degree centrality based (Das and Sinha 2016) to more complex network disruption strategies (Bruns 2011; Sevastopulo and Dyer 2015; Gerdes 2015; Reid et al. 2014). This is the first study to design and propose network disruption strategies for users’ dissemination of drive-by download attacks to the best of our knowledge. Therefore, the proposed network disruption strategies are analogous to a large body of work that has successfully undertaken a similar approach in other areas (Giommoni et al. 2021; Musciotto and Miccichè 2022; Hsieh et al. 2014). For example, Musciotto and Miccichè (2022) use a similar approach to test the effect of different network parameters (degree and betweenness centrality) to disrupt mafia groups while (Hsieh et al. 2014) and colleagues adopt the same methodology to determine the best strategy to inhibit contagion.

### Contribution

This study develops and evaluates the first approach to reducing malicious URL propagation on OSN. The study aims to identify the best network disruption strategy so that critical users posting malicious tweets can be identified and removed. Disruption strategies are based on network characteristics such as centrality, and—for the first time in the network disruption literature—we use emotion language in Twitter posts and user account characteristics as part of the disruption strategy. This paper contributes to the broader literature on malware propagation by:

- Understanding the dynamics of the interaction between disruption and resilience of networks sharing malicious URLs being propagated on OSNs;
- Simulating the effects of different disruption strategies in order to find the most efficient intervention at limiting the spread of malware on OSNs.

## Data collection and processing

### Data collection

Popular events, in particular sporting events, are known to attract a large number of active users on OSNs. These events are being used to carry out drive-by download attacks (Javed et al. 2020, 2018)—luring users to malicious webpages via shortened URLs, where the endpoint web address is not visible to the user. Furthermore, in 2018, Symantec reported in their Internet Threat Security technical report that an average of 953,800 web-based attacks occurred every day (Corporation 2019) and 1 in 10 URLs point to a malicious web server. Similarly, paloalto (Apr 2021) reported in their Threat report that trending events like ‘COVID-19’ had been used to redirect users to malicious pages, and in a span of seven weeks, around 86,000 newly observed hostnames related to COVID were identified as malicious. Considering the number of active users on OSN, the popularity of the event and the frequency of attacks, a post containing a malicious URL has the potential to expose malware to millions of users within a matter of minutes (Sanzgiri et al. 2012).

Over two years, 3,285,108 tweets containing URLs were captured from three sporting events organised in three different continents (North America, Europe and Australia). The resultant dataset comprises a geographically separated diverse set of users. Data for our study were collected by using event-specific hashtags from Twitter via the streaming API using the python library Tweepy (Roesslein 2018) (see Table 1). Though the data presented in the study is not current, it still reflects the method by which a drive-by download is launched and propagated by means of retweeting on Twitter (Horawalavithana et al. 2021; Roy et al. 2021). The three popular events that were chosen are as follows:-

1. *The American Football Superbowl 2015*: The championship game held between *New England Patriots* and *Seattle Seahawks* to determine the champion for the 2014 season of the National Football League (NFL). During the event, a total of 28.4 million tweets were recorded on Twitter.
2. *The Cricket World Cup 2015*: The international championship of *one day cricket* between 14 countries that Australia and New Zealand jointly hosted. During the event, 3.5 million tweets were recorded on Twitter (Khatua and Khatua 2017) making it one of the most popular sporing events of 2015. The data were collected for seven days starting on 24th March (the day the first semi-final between South Africa and New Zealand was played) and ending on 31st March (two days after the final between New Zealand and Australia).
3. *The European Football Championships 2016*: was the $15^{th}$ International men’s football championship of Europe organised by The Union of European Football Associations (UEFA). It was held in France from 10 June to 10 July 2016, and a total of 24 teams participated. During the event, England vs Iceland was reported to be the most-tweeted-about match, and generated 2.1 million posts during the game (Rogers 2016). Tweets containing event-specific hashtags and URLs were collected during the entire month the sporting event took place, starting from 10th June.

The reason for selecting these sporting events was to determine whether our network disruption strategy generalises beyond a single sporting fixture.

**Table 1:** Number of Tweet’s containing malicious link captured for each sporting event

Sporting event	Year	Hashtag used	Malicious tweet identified
Cricket world cup	2015	#CWC15	4,238
European football championship	2016	#Euro2016	21,559
Superbowl	2015	#SB50 #SuperBowlSunday #superbowlXLIX	2,293

### Data processing

A sub-sample was randomly created from the collected dataset in which tweets were processed to remove duplicates (retweets), and URLs were then extracted. These URLs were passed into a high interaction honeypot to identify malicious endpoints. Capture HPC (C. Seifert 2017), an open-source high interaction honeypot, was chosen for our experiment because of the transparency it provided in classifying a URL as malicious. Capture HPC has a client–server architecture, where the server instructs the client machine to visit a webpage for a specified period of time. For our experiment, an observation time of five minutes was allocated to each client machine to record changes made during the visitation. Capture HPC determines whether the URL could be classified as malicious by analysing changes made to the registry, process and file of a client machine at the end of the observation time (Puttaroo et al. 2014).

**Fig. 1:**
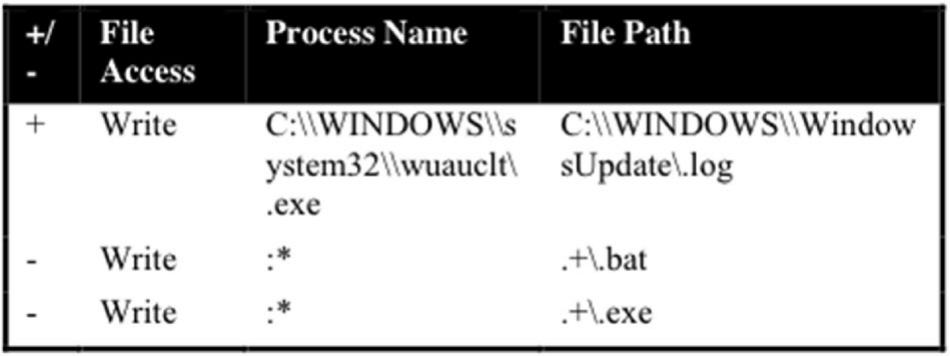
File exclusion list

A URL is classified as malicious if any rule in the three *exclusion lists* is violated. The three *exclusion lists* contain entries that distinguish benign from malicious activity while visiting a webpage. For example, a typical file exclusion will represent a malicious activity by creating a rule that prohibits any modification to executable files during the visitation of a webpage. All prohibited activities start with a negative symbol, and those activities that are acceptable begin with a positive sign (see Fig. 1).

**Fig. 2:**
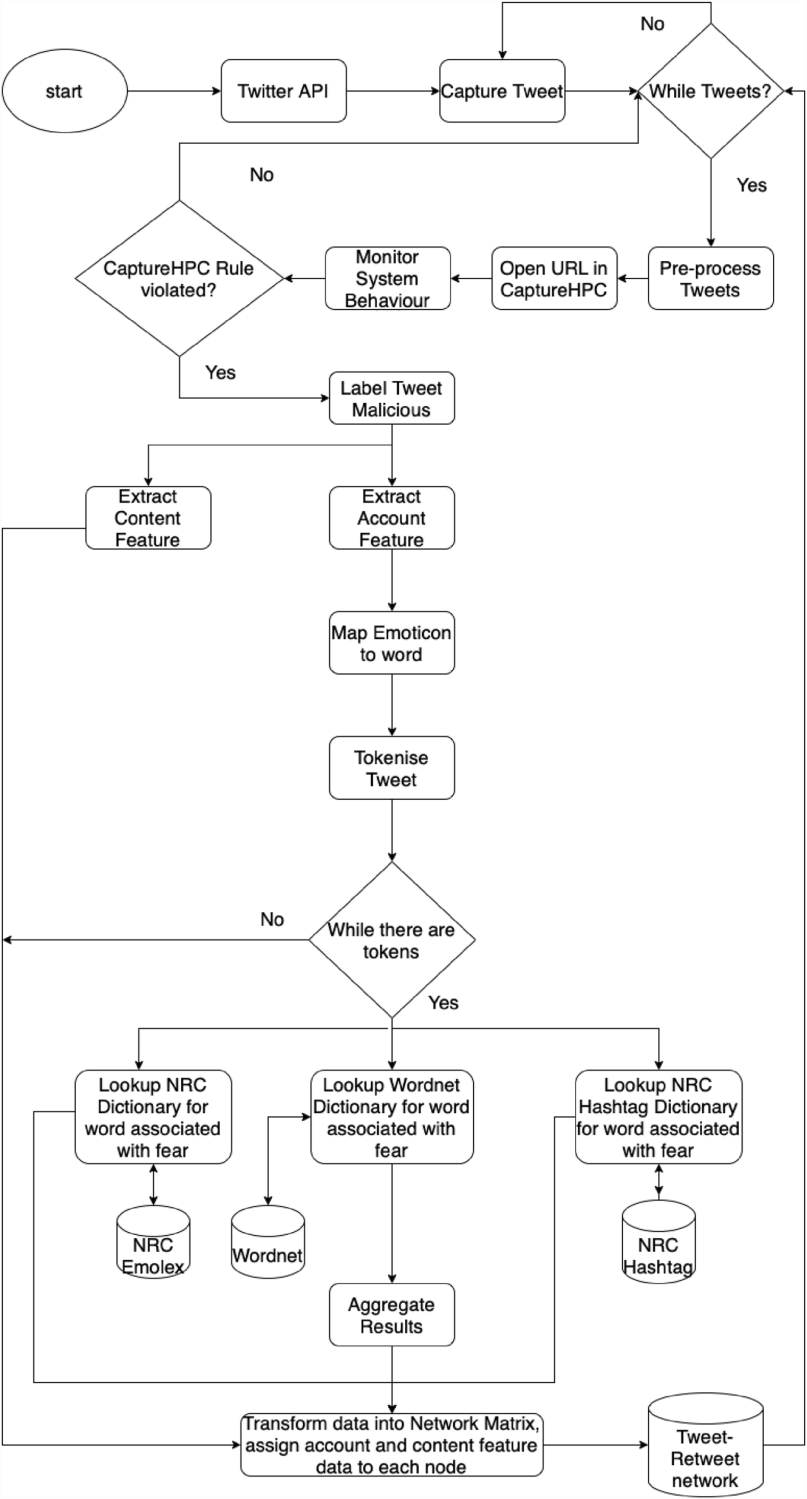
Flow chart for processing tweets and creating Tweet–retweet network

Figure 2 provides an overview of the experimental setup in the form of a flow chart. First, the program retrieved the tweets captured from the Twitter streaming API and checked whether the embedded URL was pointing to a malicious web server. This was done by opening the URL inside Capture HPC and observing changes made during the webpage visitation. Once the URL was identified as malicious, the tweet was further processed by tagging it into a tweet or retweet category. If it was a retweet, then information related to the original tweet was retrieved and added. This was done by searching through the dataset by filtering all tweets containing *RT* as the prefix.

The program then extracts and tags each tweet with associated content and account-specific features, as these are parameters used to disrupt each malicious network. For example, for account-specific features, we pull the number of followers from the user that posted the tweet, and for content-specific features, we looked at the presence of negative emotion, particularly fear.

A Java-based script that carried this out was developed to extract the emotion of fear from each tweet. The script identifies the emotion based on dictionaries containing words associated with fear. The three dictionaries that were used were built using the WordNet Affect Lexicon (Strapparava and Valitutti 2004), NRC-Emolex and the Hashtag Emotion Corpus (Mohammad and Turney 2013; Sobhani et al. 2016). In addition to these dictionaries, we used emoticons contained in a tweet.

Based on dynamic changes made to a client machine and by the violation of rules defined in the exclusion list, a total of *28,090* tweets containing malicious URLs were identified over the three sporting events and were retained for the next stage. The study aims to identify pivotal users in propagating malware by disrupting these malicious networks based on different disruption strategies. For example, even if one user sends 99.9% benign tweets and just one malicious tweet, they are labelled as a malicious user and part of a malicious network and needs to be removed. This malicious user within the network could hold a strategic position in causing maximum impact by exposing a maximum number of users to malware. In the last stage, nodes that represented tweets were created, and meta-information were mapped to them. The meta-information contained information about the number of words associated with fear, the number of followers of the user account that posted/retweeted the post, whether it was a tweet or a retweet, and if a retweet, the address of the original post.

### Tweet–retweet network formulation

Studies show that online communications can influence individual behaviours, so it is essential to understand how the information is disseminated within the network and who the influential or pivotal users are in spreading the information. This is vital to understanding online communities and how they can influence online and offline behaviours. For instance, how does information flow within a specific online community? Who are the central actors in the network? How dense is the network? Are there any subgroups within the online community? These sociometric analyses can help us understand the network and provide key information for devising strategic interventions. For instance, they can help to identify which sets of nodes would better disseminate tweets with malicious URLs within the network. As such, social media network analysis is essential for devising tailored and effective interventions. Among the many metrics present to evaluate the network’s structural characteristics, we considered the most popular ones. These are Assortativity, mean degree, density, degree centralisation and giant component to evaluate the structural characteristics of the network and are as explained below.

*Assortativity* is the tendency of vertices with a similar number of links to preferentially associate with each other (Newman 2003). It refers to the correlation of the number of links that any two connected individuals have. A positive assortativity value indicates that active or popular users tend to retweet or be retweeted from users that are also popular or active. Thus, for a network of users posting malicious URLs, a positive assortativity will indicate that users with similar links are more likely to retweet the malicious tweet.

*Degree centrality* is defined as the number of links incident upon a node, that is, the number of ties that a node has. For our network, it is the number of retweets a tweet received. A node would have a high degree of centrality if the tweet posted by the user represented by the node received a high number of retweets and vice versa if there were fewer number retweets. This measure is used to identify nodes (users) that have a high number of users linked to them.

*Betweenness centrality* measures the number of times a node is along the shortest paths between any two other vertices in the network. This measure is often used to identify a central user and have a high probability of exposing malware to a maximum number of users in the network.

*Giant component* is the most significant connected subset of vertices in the network. We also calculate the average geodesic distance, which is the mean shortest path between any two vertices, and the diameter. One of the main properties of a giant component is that its size grows linearly with a number of nodes and, in our case, number of users posting malicious posts.

*Mean degree* is simply the average number of edges per node in the graph. We calculate these by dividing total edges by total nodes. This statistic informs us that users of the malicious network have more users retweeting the malicious post. We are thus able to gain insight into the causes and/or underlying conditions that shape the network comprising of users posting malicious posts. An information which is useful in designing network disruption strategy so that users can be effectively removed to stop malware propagation.

*Density* is a measure of how many ties between users exist compared to how many ties between users are possible. The Density of a network property is important to consider for mainly two reasons. First, it can help us understand how connected the network is compared to how connected it might be. Second, when comparing two networks with the same number of nodes and the same type of relationships, it can tell us how the networks are different. It helps understand information flow within the network if one or two users are removed. Also, the density of sub-graphs within the network is used to examine the array of subgroups within a broader organisation.

*Krackhardt’s connectedness score* is equal to the fraction of all dyads connected through an undirected path (Krackhardt 1994). It is equal to the fraction of all pairs (user that tweeted (i) and user that retweeted (j)), such that there exists an undirected path from i to j in a malicious tweet–retweet network. Values of connectedness close to 1 indicate that all nodes (tweeted and retweeted) are connected with every other node in the graph, while values close to 0 indicate that the graph’s nodes are isolated. We use Krackhardt’s connectedness score to evaluate how connected the malicious network is? The more the score value closer to zero, the higher the number of isolated users and fewer chances of malware propagation within the network.

**Fig. 3:**
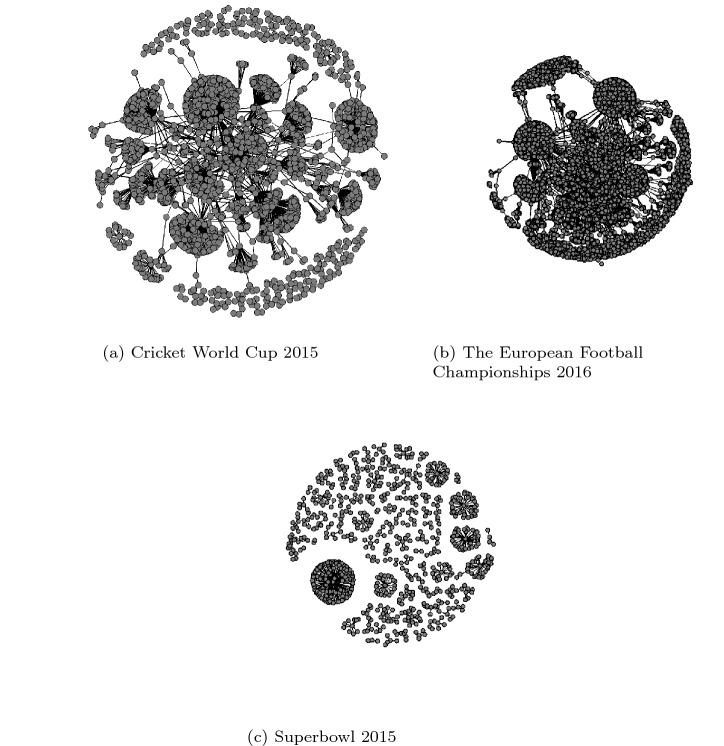
Tweet–retweet network

*Tweet–retweet network*– After processing and annotation of tweets, we created a tweet–retweet network (see Fig. 3), where each node *N* is represented by a user that either posted/retweeted a malicious tweet and an edge between two nodes, $N_a$ and $N_b$ existed if $N_b$ retweeted $N_a$ tweet. Where a malicious tweet is a tweet containing an URL pointing to a webpage containing harmful code/program. Each node was identified by analysing the text within the tweet and machine behaviour of the system on which a Web page was opened. The Web link within the text and observation of system behaviour while visiting the Web page defined the maliciousness or otherwise. Therefore each user within the network is a presentation of the tweet and system behaviour. The network consists of the user that posted malicious tweets only, as the focus of the research is to understand the disruption of the malicious network. Furthermore, for each network, we then quantified network parameters using social network analysis measures. All our analyses were performed by creating an ‘R’ script using ‘SNA’ libraries (Butts 2020). Table 2 gives a summary of the main structural characteristics we identified of the tweet–retweet networks of users posting content containing malicious URLs.

In the last part of the analysis, we sought to quantify how connected the users posting malicious tweets are with other users retweeting those posts, to understand the node removal strategy’s impact. For this, Krackhardt’s connectedness was chosen because of its popularity in measuring the spread of a disease in a network (Moustakas and Evans 2017; Giommoni et al. 2020) and, in this case, measuring the spread of malware in the social network. While evaluating each network disruption strategy, Krackhardt’s connectedness score was calculated using the ‘connectedness’ function in the ‘SNA’ package each time a node was removed from the malicious graph (Butts 2020). While a node removal strategy aims to disrupt the information path, information cannot flow from one user to another. Krackhardt’s connectedness score calculated after removing a node tells us whether information can easily flow (score closer to one) and whether no information can flow (closer to zero) as the network consists of only isolated nodes.

An effective network disruption strategy is therefore one that brings Krackhardt’s connectedness score close to ‘zero’, removes giant components, and reduces the density of the network by removing the least number of nodes.

### Node removal strategies

Five node removal strategies were designed to disrupt the network of users posting tweets containing malicious URLs. The network disruption strategies can be broadly categorised into: (i) based on network characteristics, (ii) based on user account characteristics and (iii) Tweet content characteristics. In addition to these, we have evaluated a strategy by randomly removing nodes to disrupt the network. Our decision to base the node removal strategy on these parameters is grounded in the literature about criminal networks (Musciotto and Miccichè 2022; Giommoni et al. 2021), transmissible infections (Giommoni et al. 2021; Rocha et al. 2010) and malware distribution (Javed et al. 2020; Sanzgiri et al. 2012; Wang et al. 2016). In designing node removal strategy based on network characteristics, we looked at degree and betweenness centrality. Degree centrality measures the number of users with which each user node is connected to via a malicious connection. Betweenness centrality, instead, measures the number of times a node is along the shortest path between any other nodes in the network (represented by user posting/retweeting malicious tweet). This measure is often used to identify central users that are strategically positioned in the network to help disseminate information; that is, they facilitate the exchange of information within the network. From an epidemiological perspective, these parameters can be helpful in identifying individuals who are likely to play critical roles in the dissemination of information (Wasserman et al. 1994; Hsieh et al. 2014).

While designing a network disruption strategy based on content, we designed a disrupting strategy by removing nodes based on the emotion of fear found in the tweet content. The emotion fear was chosen because previous research show news that contains negative emotion such as fear was more likely to be retweeted than actual stories that reflected anticipation, sadness, joy and trust (Vosoughi et al. 2018). Furthermore, evidence finds malicious tweets containing negative emotion, particularly fear, were more likely to be retweeted by 114% than those containing positive emotions (Javed et al. 2020). The network disruption strategy based on account characteristics was designed based on the number of followers. It was chosen because it determines the least number of people that are exposed to malicious posts. Furthermore, earlier works have demonstrated that the number of users being infected by malware is dependent on the number of followers an account has Sanzgiri et al. (2012). The five node removal strategies that are designed to disrupt the malicious network are as follows:-

**Fig. 4:**
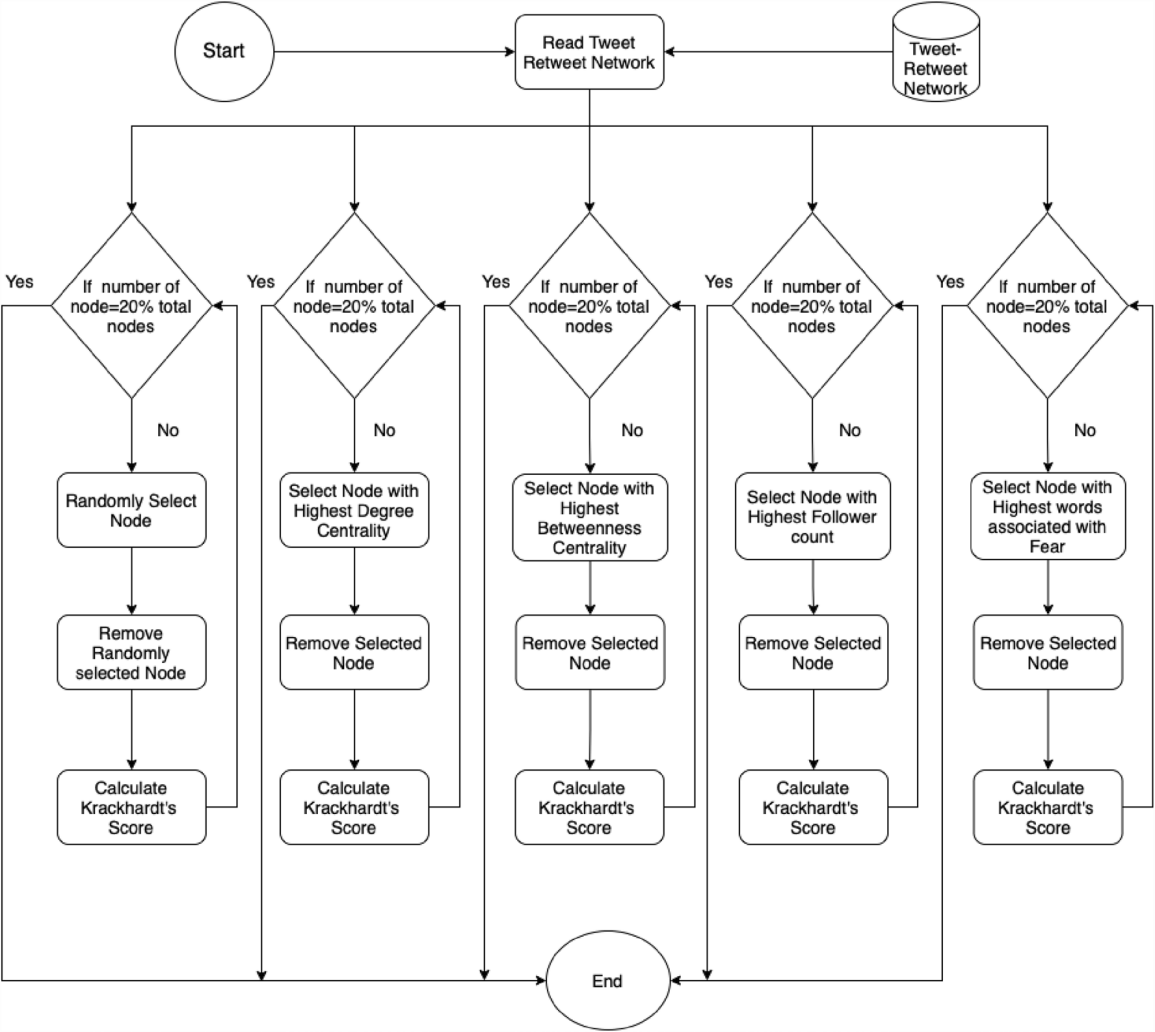
Nodes removed based on five strategies

1. Randomly removing nodes - Nodes in this strategy were randomly selected and removed (see Fig. 4). The nodes removal strategy was used to create a baseline to evaluate the performance of other network disruption strategies.
2. Based on the degree centrality score of each node - In this strategy, nodes that had the highest degree centrality were removed first (see Fig. 4), and after each removal, Krackhardt’s connectedness score was calculated to evaluate whether a retweet of a malicious tweet can expose other users to malware and thus propagate malicious tweets over the network.
3. Based on the betweenness centrality score of each node - Similarly, in this strategy, nodes that had the highest betweenness centrality were removed first (see Fig. 4).
4. Based on the number of followers each user has - Similar to other strategies, in this strategy nodes that had the highest number of followers were removed first (see Fig.  4). The rationale of including this strategy is to disrupt the network by limiting the exposure (malicious tweet is seen by followers of a user posting the tweet) and its propagation (malicious tweet–retweet networks represent how malware spreads).
5. Based on the emotion fear - In this node removal strategy, nodes that posted a tweet containing the highest number of words associated with the emotion fear were removed first (see Fig. 4). This strategy removes those users who are using fearful tweets to increase the retweetability of malicious posts (Javed et al. 2020; Berger and Milkman 2013, 2012; Vosoughi et al. 2018).

The five resulting removal strategies approximate the impacts of different immunisation strategies which might focus on individuals with a specific role or position within the network. Each of the five strategies mentioned above were applied to all the three tweet–retweet networks of malicious users.

## Results and discussion

**Table 2:** Structural Characteristics of the Tweet–Retweet Network

Dataset	Nodes	Edge count	Density	Mean Degree	DC	Assort.	GCV	GCE	AGD	DGC)	MDC
Cricket 2015	2183	2270	0.00095	1.03985	0.137	-0.030	2059	1890	4.7	11	302
Euro 2015	12942	13478	0.00016	1.04142	0.216	-0.061	11685	12508	4.2	15	2796
SuperBowl	794	664	0.00211	0.83627	0.157	0.017	126	127	1.9	2	126

*GCV* Giant component vertices, *GCE* Giant component edges, *AGD* Average geodesic distance (in GC), *DGC* Diameter in GC, *MDC* Max degree Centrality, *DC* Degree centratization, *Assort* Assortativity

A maximum of 12,942 nodes and 13,478 edges and a minimum of 794 nodes and 664 edges (see Table 2) were recorded in the three malicious tweet–retweet networks. Each node represented a user that either posted a tweet containing a malicious URL, or retweeted a post containing a malicious URL. An edge that connected the two users described a tweet–retweet relationship, i.e. two users were connected if one of them had retweeted the tweet or retweet posted by the other.

We identified a giant component in each of the three networks as a subset of the malicious network where users are all linked together. We found that 90% of users in the European football championship and 94% in ICC Cricket world cup were part of the giant component. In contrast, only 15% of users were part of the giant component in the Superbowl network (see Table 2). This suggests, with an increase in the number of users in the malicious network for Euro 2015 and ICC 2015, the size of the giant component would grow linearly, whereas that will not be the case in the Superbowl network. From a disruption perspective, the strategy should prioritise giant components first before targeting other areas in the network. The users who were not part of the giant component should not be prioritised as they were either component or isolated, and as such have little impact on malware propagation. An isolated node (user) or component was disconnected from the main network and has no impact on information dissemination within the network. For the Superbowl network giant component, the average geodesic distance, representing the shortest path between any two vertices, is relatively short. On average, a user can reach any other user in the giant component using one intermediary (or two steps). The average geodesic distance for the European football championship and ICC Cricket World Cup network is 4. (A user can reach another user using three intermediaries.) Considering the number of users in a giant component and average geodesic distance, a cybercriminal could spread malware faster in a network with a small distance, as it will take less number of hops to reach all the users. For example, by targeting high profile accounts such as Joe Biden, Barack Obama, Mike Bloomberg that tend to form Giant components within Twitter, cybercriminals were able to expose a large number of users to a cyberattack strategically and were successful in stealing around $120,000 within two hours of compromising these high profile accounts (M 2020). Thus, a node removal strategy would be more effective by reducing the propagation rate by strategically removing nodes along the shortest path, so malware cannot propagate quickly.

In our study, in two networks, the European football championship and the Cricket World Cup, the assortativity score is negative (-0.03 and -0.06, see Table 2) and positive for the Superbowl. An assortativity score measure similarity of a user in terms of the number of edges attached to them, which in our study reflect the number of retweets. A negative assortativity score indicates relationships between nodes of different degrees (number of connections each node has). Where a number of connections each node gets are dependent on a retweet, which is driven by content of posts (such as emotion) (Javed et al. 2020) or account characteristics of a user posting the tweet (Javed et al. 2020; Sanzgiri et al. 2012; Javed et al. 2018; Lee and Kim 2013). The positive assortativity (0.0017) recorded for the Superbowl may indicate homophily or assortative mixing, which is edge formation due to similarity in node characteristics, such as the number of followers a user has. A malware propagation response strategy should tackle both types of networks (assortative and disassortative) by designing a strategy that focuses on the characteristics of a node and factors that help in the formation of links (tweet–retweet relationship) between users.

### Node removal to model malware propagation response strategies.

The highest overall connectivity among the three networks was found to be 0.8152 in Euro 2016 event. 81.52% of all dyads are connected through an undirected path in the malicious network. Whereas for the ICC network, it was 0.74, and for Superbowl, it was 0.0372, making Euro 2016 the most connected and Superbowl the least connected network. Network connectivity determines how easy it is for nodes (users) to interact with each other. The higher the network connectivity, the easier it is for nodes to interact with others as a path exists between users and vice versa. From a malware propagation perspective, in a highly connected network, it will be much easier and quicker for a cybercriminal to infect many users compared to a network with low overall network connectivity.

Each time a user retweets a malicious post, the malware is propagated within the network and is exposed to more users (followers of the person retweeting the post). The study aims to strategically remove the minimum number of users (nodes) to reduce network connectivity and curb the malware propagation/exposure in a network. Five different strategies were applied to identify the most effective way to decrease network connectivity by removing the minimum number of users. These include removing nodes based on their degree, betweenness centrality scores, number of words associated with the emotion fear, number of followers a user has, and randomly selecting a user to remove from the network.

**Fig. 5:**
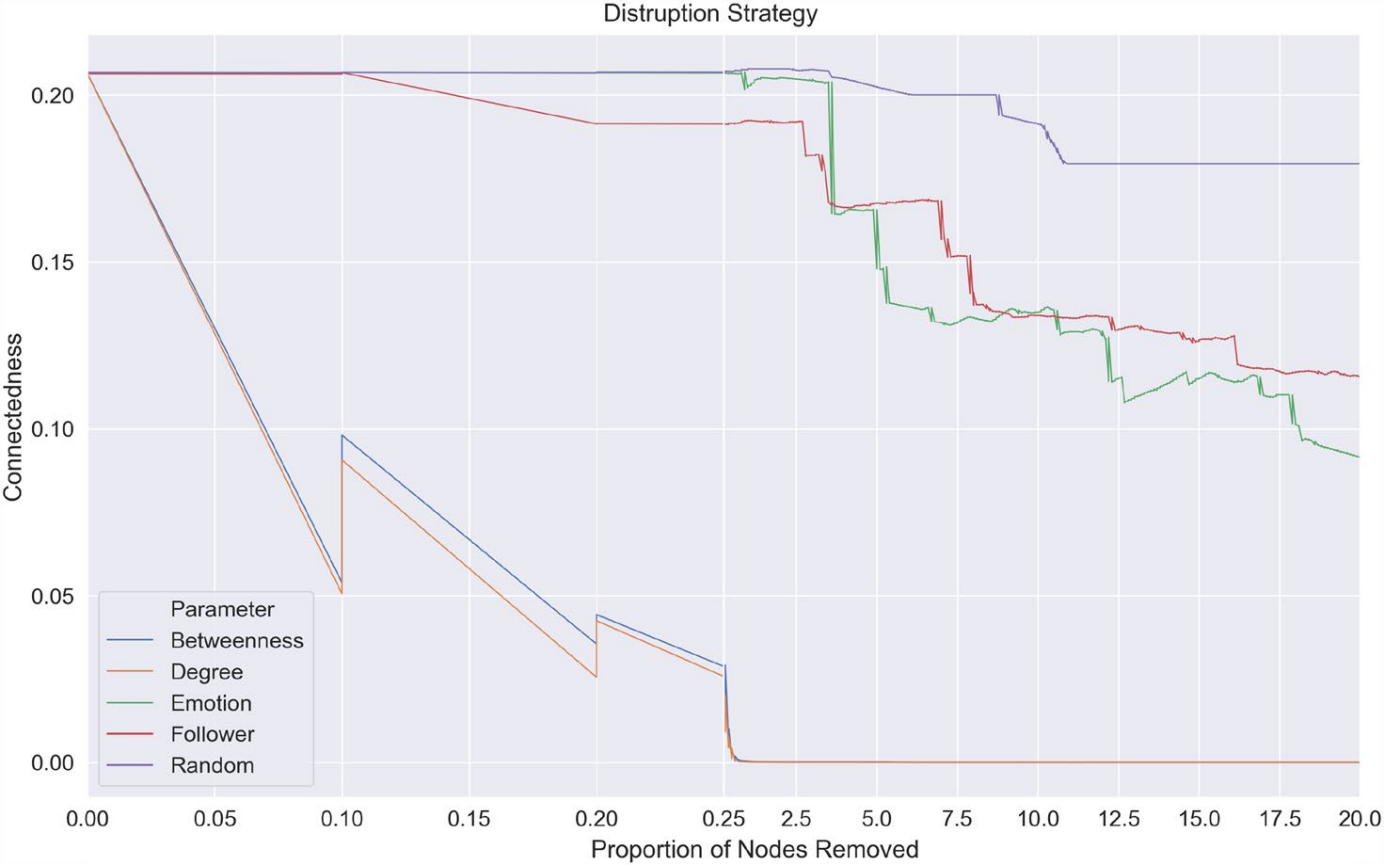
Node Removal Strategies on Cricket World Cup 2015 malicious network

**Fig. 6:**
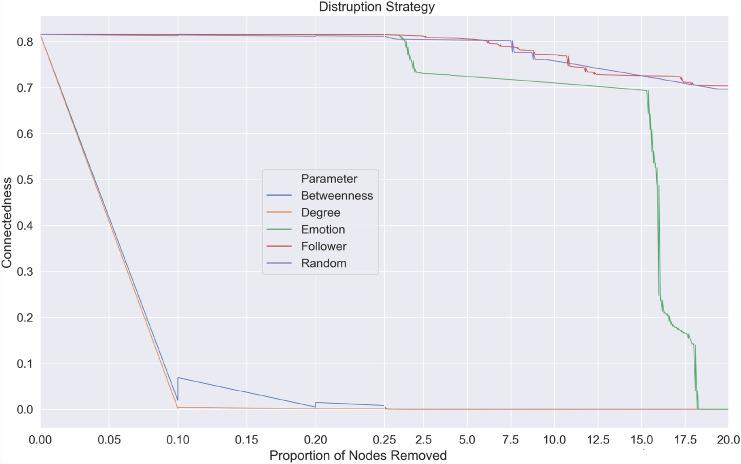
Node Removal Strategies on the European Football Championships 2016 network

**Fig. 7:**
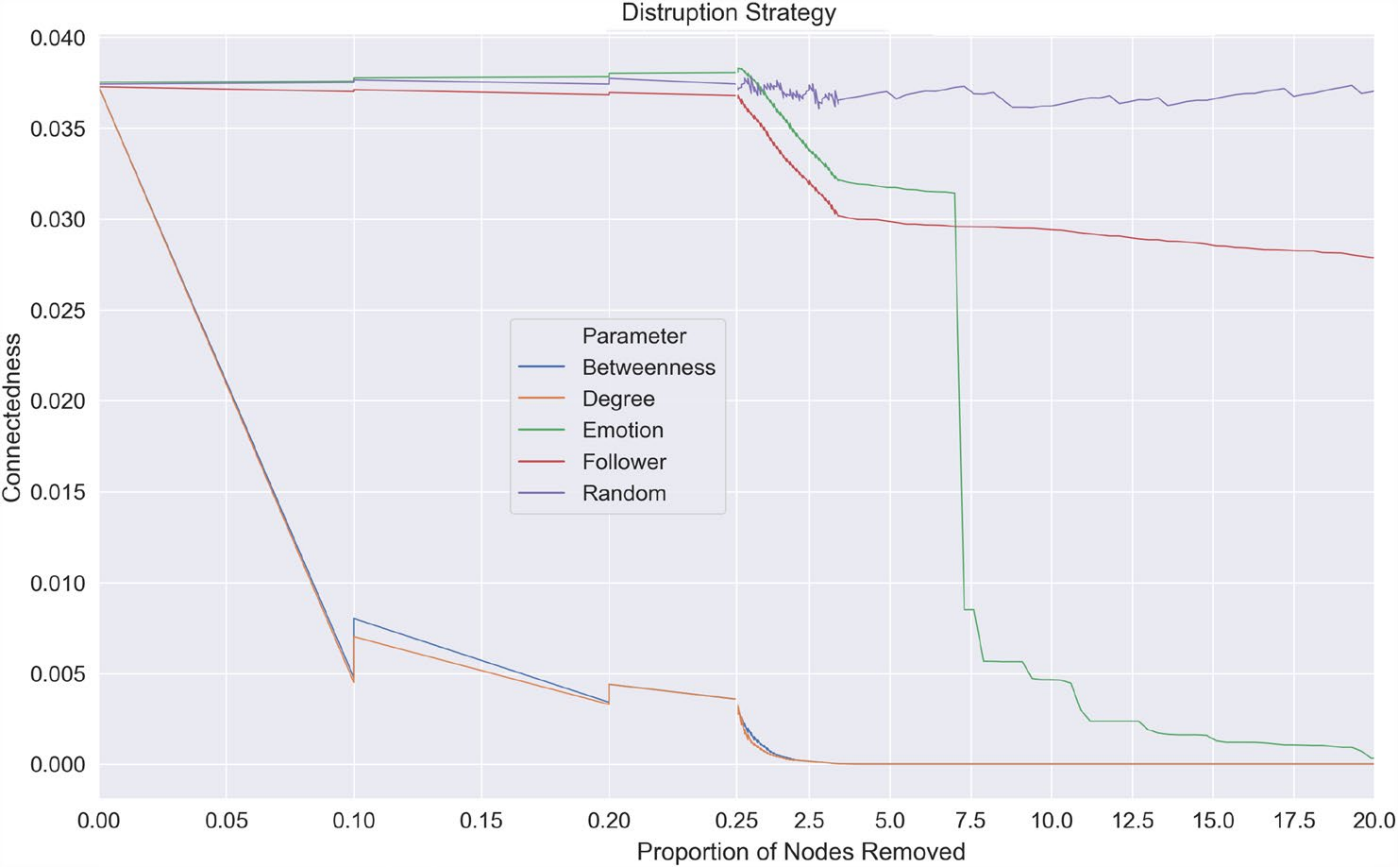
Node Removal Strategies on the Superbowl 2015 network

Out of the five node removal strategies tested on three different malicious networks, the random node removal strategy was the least effective. For any proportion of nodes removed using the random strategy, all three networks’ connectedness remained relatively unaffected when compared to the more targeted strategies (see Fig. (5,6,7)). Interestingly, the node removal strategy based on the number of followers was least effective among the targeted interventions. This could be because nodes with a high number of followers were not well connected with other nodes. As two of the malicious networks exhibited disassortative (ICC and Euro) characteristics and one assortative (Superbowl) characteristics, malicious networks are formed by user interactions that may or may not be similar. Results from disruption strategy based on followers of a user showed that popular accounts, even though they can expose many people if removed, have little impact on the diffusion of malicious links.

The removal of nodes based on content, i.e. the number of words associated with fear, performed better in all networks than node removal strategies based on the number of followers and random removal. The connectedness drops from 0.80 to 0.01 after removing 18% of ‘fear’ posting nodes in the Euro 2016 network, from 0.03 to 0.008 in the Superbowl network after removing 7% of nodes, and from 0.21 to 0.10 after removing 12% of nodes in the Crick World Cup network. The findings are in line with earlier work, where negative emotions such as fear played a stronger role in spreading malware (Javed et al. 2020) or information (Vosoughi et al. 2018; Berger and Milkman 2012, 2013) than the number of followers (Javed et al. 2020).

Removal strategies based on network characteristics such as degree centrality and betweenness were the most effective, where a substantial decrease in connectedness is seen in all three networks. Where betweenness represents the degree to which users posting malicious URLs stand between each other, and connectedness represent the number of links (retweets) a malicious URL receives. This finding suggests that disrupting malware propagation by removing nodes (users) that lie along the shortest path or those nodes with a large number of users connected to them was more effective than those based on content or account characteristics. Out of the five node removal strategies tested on three different malicious networks, the random node removal strategy was the least effective. For any proportion of nodes removed using the random strategy, all three networks’ connectedness remained relatively unaffected when compared to the more targeted strategies (see Fig. (5,6,7)).

**Fig. 8:**
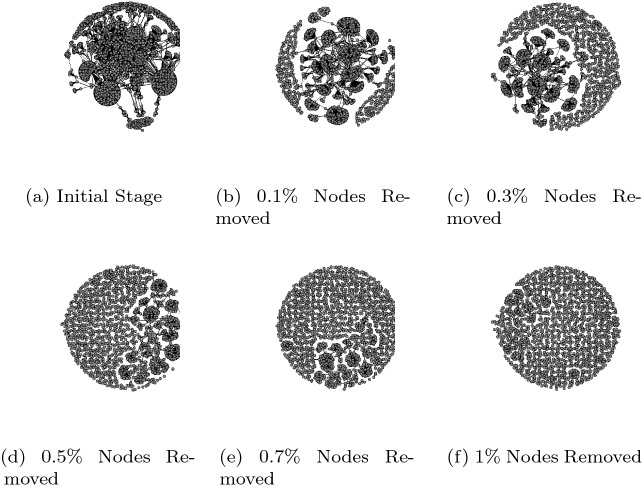
Impact of Node removal based on degree centrality on the European Football Championships 2016 malicious network to model malware propagation response

We know the established factors that help OSN network propagation (Javed et al. 2020; Berger and Milkman 2013; Sanzgiri et al. 2012). However, these factors do not account for the connectivity of the network and may not be effective vectors for disruption. For example, an isolated node with a high number of followers posting a malicious tweet has more chance of being retweeted, but due to its position in the network, if removed, it has little impact in terms of overall disruption. However, by removing nodes that have a high degree of centrality or betweenness, the propagation of malware is disrupted by disconnecting paths between users so infection can be isolated (see Fig. 8). The strategy based on degree centrality was slightly more effective than betweenness centrality, as after removing 1% of nodes in all the three networks, the connectedness dropped below 0.05. This suggests a more significant impact is created by removing nodes that have a high number of users attached to them than those nodes that lie along the shortest path in the malicious network. The results showed that by removing these highly connected users, one could successfully disrupt the malicious network (see Fig. 8) by isolating the infected nodes from the network so that each user is disconnected and cannot further infect another user. Combining these best performing network disruption strategies with the less effective, but novel removal strategy based on locating fear words in post content, will achieve the optimum outcome.

### Real-world use case

**Fig. 9:**
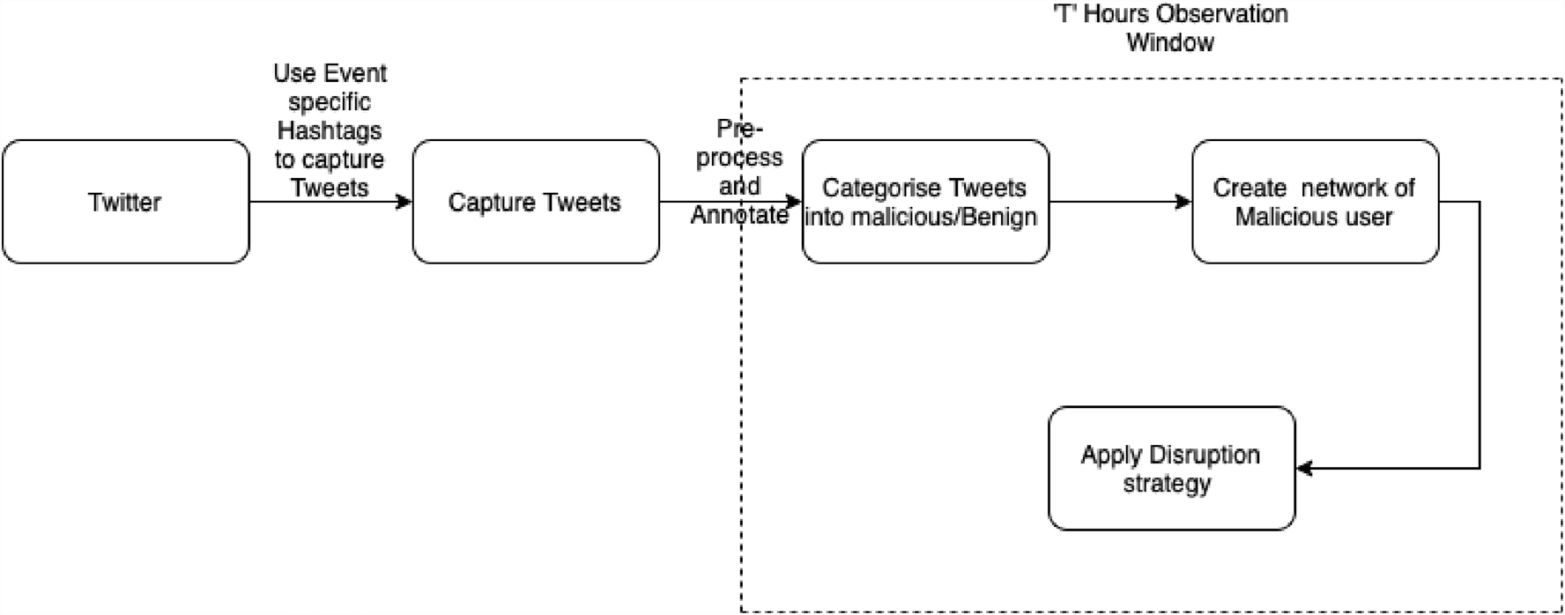
Experimental setup to deploy network disruption strategy

To demonstrate the deployment of a network disruption strategy, we present a use case to contextualise how the network disruption strategy is applied in the real world. Considering popular sporting events are used to launch and propagate drive-by download attacks (Javed et al. 2020). Tweets containing URLs around popular events, preferably sporting events, are captured using event-specific hashtags (refer to Fig. 9). An observation window of ‘T’ hours is defined within which tweets are processed and annotated into malicious or benign using Capture HPC (Javed et al. 2018; Burnap et al. 2014). For a more detailed explanation of the processing and annotation of tweets, refer to sect. 3.2 and Fig. 2.

Once the tweets are processed and annotated, a network of users posting malicious tweets is created. Among the different strategies, the one based on degree centrality is the most efficient at disrupting the spread of malicious links (refer to sect. 4.1). For a more detailed explanation of node removal based on degree centrality, refer to sect. 3.4 and Fig. 4. The experimental results have shown that only 1% of nodes need to be removed to make the network disconnected. Thus, stopping malware from being propagated within the network. The application of the network disruption strategy in the observation window ensures that malicious users are identified and are strategically removed from the network before they can disseminate malware within the network. The intervention could be a cyclic process in which a network disruption strategy is applied every *T hours* to identify and remove malicious users strategically. The static graph created by defining the time for the observation window ensures that malicious users in strategic positions to launch an impactful attack are removed first from the network. Thus, intervening at the early stages of the malware propagation.

## Conclusion

This study collected datasets from three different sporting events and identified tweets containing malicious URLs. A URL was classified as malicious if a drive-by download attack occurred while visiting the Web page. From the tweets/retweets identified as malicious, a tweet–retweet network was created representing the diffusion of malicious URLs. Using the three malicious tweet–retweet networks, we conducted the first social network analysis to identify the most effective strategy to disrupt the spread of malicious codes on social media. We formulated content, user account, and network-based strategies to identify the most effective in decreasing a network’s connectedness to curb malware propagation. For the content-based strategy, we removed nodes based on words in the tweet/retweet that were associated with fear. We removed nodes in account-based strategies based on number of followers. Finally, we removed nodes based on degree centrality and betweenness centrality for strategies based on network features.

Our analysis on all three networks showed that interventions based on network features are the most efficient to quickly reduce a network’s connectedness. Specifically, the results show that removing 0.5% of nodes (i.e. 641 nodes in Euro 2016, 39 nodes in Superbowl and 109 nodes in Cricket World Cup) based on degree centrality is 20 times more effective than randomly removing 20% of nodes. Simultaneously, strategies based on account features, such as a number of followers and or emotion-related contents, are more effective than random intervention but less than network-based interventions.

This is the first time that a study has shown that targeted malware network disruption interventions are more effective than the random removal of accounts in OSN. As already shown in the analysis of offline sexual and criminal networks, while many nodes composed these networks, only a few of them play a key role in the overall structure of these networks. We can drastically alter these networks, and the negative consequences associated with them, by identifying these key players, instead of randomly attacking the entire network. From this perspective, this study offers a tool for identifying the key players involved in the spread of malicious codes on social media. This could be useful for security experts in their efforts to be more efficient and cost-effective in limiting the spread of malware. Better results can be obtained by using fewer resources on a restricted and targeted number of key players.

## Limitation

Our work is not without limitation. Although we study different malicious networks in detail, we worked with a scarcity of baseline datasets. One of the biggest challenges in identifying and annotating tweets as malicious quickly before they disappear is tackling the rate at which the tweets are posted (6000 tweets per second (MohamedSikandar 2018)) against tweets processed per day (around 1000 tweets (Javed et al. 2018)) using a high interaction honeypot. In future, we may see a scalable detection and annotation model that could match the speed of users posting tweets. The current research focuses on disruption strategies on a static graph, representing a formed network of users tweeting or retweeting over different periods of time. The study aimed to identify pivotal users in propagating malware by disrupting these malicious networks based on different disruption strategies. However, once a user is removed dynamically from the network, a network can reform and evolve. In our future work, we will simulate the reformation of the network once a user has been removed and continue with the node disruption strategy to identify which works best over evolving networks. Furthermore, we aim to develop and apply more complex network disruption strategies based on users tweeting/retweeting behaviour. Where node selection for removal could be based on the user’s frequency of posting/sharing malicious links. So that those users exhibiting more malicious behaviour are removed before those exhibiting less.

## Data Availability

The datasets generated during and/or analysed during the current study are not publicly available as our ethics policy does not allow data containing personal information to be published, given it is now labelled as ‘malicious’ and an ‘users’ information can be retrieved using the tweet’s content.
